# Identification of Reduced Circulating Haptoglobin Concentration as a Biomarker of the Severity of Pulmonary Embolism: A Nontargeted Proteomic Study

**DOI:** 10.1371/journal.pone.0100902

**Published:** 2014-06-30

**Authors:** María Insenser, Rafael Montes-Nieto, M. Ángeles Martínez-García, Elena Fernandez Durán, Carmen Santiuste, Vicente Gómez, Jeffrey A. Kline, Héctor F. Escobar-Morreale, David Jiménez

**Affiliations:** 1 Diabetes, Obesity and Human Reproduction Research Group, Department of Endocrinology & Nutrition, Hospital Universitario Ramón y Cajal & Universidad de Alcalá & Instituto Ramón y Cajal de Investigación Sanitaria (IRYCIS) & Centro de Investigación Biomédica en Red Diabetes y Enfermedades Metabólicas Asociadas (CIBERDEM), Madrid, Spain; 2 Department of Clinical Biochemistry, Hospital Universitario Ramón y Cajal, Madrid, Spain; 3 Department of Internal Medicine, Hospital Universitario Ramón y Cajal, Madrid, Spain; 4 Department of Emergency Medicine, Indiana University School of Medicine, Indianapolis, Indiana, United States of America; 5 Respiratory Department, Pulmonary Embolism Research Group, Hospital Universitario Ramón y Cajal & Universidad de Alcalá & Instituto Ramón y Cajal de Investigación Sanitaria (IRYCIS), Madrid, Spain; Brighton and Sussex Medical School, United Kingdom

## Abstract

Risk stratification of patients with pulmonary embolism (PE) may identify patients at high risk of early death who may benefit from more intensive surveillance or aggressive therapy. Nontargeted proteomics may identify biomarkers useful for the risk stratification of patients with acute symptomatic pulmonary embolism (PE). We studied 6 patients presenting with low-risk PE and 6 patients presenting with intermediate (n = 3) or high-risk (n = 3) PE. Two-dimensional difference gel electrophoresis was used to compare their plasma protein abundances. Candidate protein markers were identified by matrix assisted laser desorption ionization time-of-flight mass spectrometry. A panel of four biomarkers (haptoglobin, hemopexin, α_2_-macroglobulin, and Ig α_1_-chain C region) showed differences in plasma abundance among patients with acute symptomatic PE of different severity. Haptoglobin and hemopexin were decreased, whereas α_2_-macroglobulin and Ig α_1_-chain C region were increased, in patients with high or intermediate-risk PE compared with low-risk PE patient. In a separate clinical population consisting of 104 adults with acute PE, serum haptoglobin concentrations had an 85% chance of correctly identifying patients with high-risk PE according to receiving operating characteristics curve analysis. Moreover, serum haptoglobin concentrations ≤1 g/l showed an 80% sensitivity and a 96% specificity for the diagnosis of high-risk PE. Nontargeted proteomics identified protein biomarkers for the severity of PE that are involved in iron metabolism pathways and acute-phase response. Among them, reduced serum haptoglobin concentrations show a high accuracy for the biochemical detection of high-risk PE.

## Introduction

Venous thromboembolism (VTE) includes deep vein thrombosis (DVT) and pulmonary embolism (PE). PE is a major cause of mortality, morbidity, and hospitalization in Europe. On the basis of an epidemiological model, it was estimated that over 317 000 deaths were related to VTE in six countries of the European Union in 2004 [1]. Of them, 34% presented with fatal sudden PE and 59% were deaths resulting from a PE that remained undiagnosed during life; of note, only 7% of the patients who died early had been diagnosed with PE before death.

Mortality rates in patients with objectively confirmed acute pulmonary embolism (PE) vary from 1.4% to 17.4% during the first three months of treatment [2], [3], [4], [5], illustrating the heterogeneous clinical and prognostic spectrum of this disorder. Acute complications (e.g., acute pulmonary arterial hypertension and right heart failure) or recurrence of PE cause most early deaths, whereas medical problems underlying PE are responsible for most late deaths [6].

The initial management of PE aims to prevent fatal and non-fatal recurrent venous thromboembolism and pulmonary arterial hypertension from PE, while minimizing treatment-related complications such as bleeding. For patients with concomitant deep vein thrombosis, treatment objectives also include the prevention of the post-thrombotic syndrome. Risk stratification of patients with PE may identify patients at high risk of early death who may benefit from more intensive surveillance or aggressive therapy (i.e., thrombolysis or embolectomy) [7], [8]. Alternatively, patients deemed low risk for early complications (i.e., death, recurrent venous thromboembolism, and major bleeding) might be considered for partial or complete outpatient treatment of their PE [9], [10].

Nontargeted proteomics may identify novel proteins associated with different disease states [11], [12], [13]. To our best knowledge, no exploratory proteomic studies of biomarkers of severity in patients with PE have been published to date. Identification of novel protein markers of the severity of PE might provide not only new mechanistic insights but, more importantly, may facilitate prospective risk assessment and risk reduction interventions.

## Material and Methods

We conducted a nontargeted exploratory screening for markers of severity in patients with acute symptomatic PE using two-dimensional differential gel electrophoresis (2D-DIGE) of plasma samples followed by matrix-assisted laser desorption/ionization–time-of-flight/time-of-flight mass spectrometry (MALDI-TOF/TOF MS). This approach allows high-throughput analysis of thousands of proteins identifying, within a single experiment, changes in abundance according to the severity of PE. The clinical usefulness of the biomarkers identified by the nontargeted proteomic experiment was then validated in a separate larger population of patients with PE presenting with different risks and severities.

### Subjects

The nontargeted proteomics discovery study involved a total of 12 patients with acute PE. Patients were recruited at the Department of Emergency Medicine of Hospital Ramón y Cajal. Eligibility for this study required that patients presented with an acute symptomatic PE that was confirmed by objective testing. Six patients presenting with intermediate (n = 3) or high-risk (n = 3) PE were compared with 6 patients presenting with low-risk PE). The groups were similar in terms of age, body mass index (BMI) and comorbid diseases such as cancer, chronic obstructive pulmonary disease, and congestive heart failure (Table 1). The biomarkers identified by the nontargeted proteomic study were then validated by targeted approaches in an independent population consisting of 104 PE recruited consecutively at the Department of Emergency Medicine. The validation study included 5 patients with high-risk PE, 47 patients with intermediate-risk PE, and 52 patients with low-risk PE (Table 2). Blood samples were obtained approximately at the time of diagnosis of PE, and never beyond the first 24 hours after diagnosis. Fasting was not required for testing. The Institutional Review Board of Hospital Ramón y Cajal approved the study and all patients provided written informed consent.

**Table 1:** Clinical characteristics of the patients included in the two-dimensional difference gel electrophoresis nontargeted proteomic marker discovery study.

	Low-risk PE	Intermediate or high-risk PE	*P*
	n = 6	n = 6	
**Clinical characteristics**			
Age, years	80.2±5.7	80.0±6.3	0.699
Male sex	2 (33%)	2 (33%)	0.999
BMI, kg/m^2^	25.4±3.0	24.0±2.7	0.405
**Risk factors for venous thromboembolism**			
Cancer*	0 (0%)	0 (0%)	-
Recent surgery†	1 (17%)	0 (0%)	0.296
Immobilization‡	1 (17%)	2 (33%)	0.505
Comorbidities			
Chronic obstructive pulmonary disease	0 (0%)	0 (0%)	-
Congestive heart failure	0 (0%)	0 (0%)	-
**Echocardiography and cardiac biomarkers**			
RV dysfunction (echocardiogram)	0 (0%)	6 (100%)	-
BNP, pg/ml	292±395	490±274	0.132
cTnI, ng/ml	0	0.12±0.12	0.065

Data are means ± SD or counts (percentage).

*Abbreviations*: BMI, body mass index; BNP, brain natriuretic peptide; cTnI, cardiac troponin I; PE, pulmonary embolism; RV, right ventricle.

*Active or under treatment in the last year.

† In the previous month.

‡ Immobilized patients are defined in this analysis as non-surgical patients who had been immobilized (i.e., total bed rest with bathroom privileges) for ≥4 days in the month prior to PE diagnosis.

**Table 2:** Clinical variables of the patients included in the clinical validation study.

	Low-risk PE	Intermediate or high-risk PE	*P*
	n = 52	n = 52	
**Clinical characteristics**			
Age, years	70.6±16.8	67.1±16.8	0.128
Male sex	21 (40%)	23 (44%)	0.754
BMI, kg/m^2^	27.8±6.7	25.9±4.1	0.252
**Risk factors for venous thromboembolism**			
Cancer*	7 (13%)	13 (25%)	0.148
Recent surgery†	4 (7.7%)	4 (7.7%)	0.977
Immobilization‡	11 (21%)	5 (9.6%)	0.094
**Comorbidities**			
Chronic obstructive pulmonary disease	5 (9.6%)	4 (7.7%)	0.704
Congestive heart failure	1 (1.9%)	2 (3.8%)	0.569
**Echocardiography and cardiac biomarkers**			
RV dysfunction (echocardiogram)	0 (0%)	52 (100%)	<0.001
BNP, pg/ml	45±23	456±800	<0.001
cTnI, ng/ml	0.001±0.01	0.15±0.26	<0.001

Data are means ± SD or counts (percentage).

*Abbreviations*: BMI, body mass index; BNP, brain natriuretic peptide; cTnI, cardiac troponin I; PE, pulmonary embolism; RV, right ventricle.

*Active or under treatment in the last year.

† In the previous month.

‡ Immobilized patients are defined in this analysis as non-surgical patients who had been immobilized (i.e., total bed rest with bathroom privileges) for ≥4 days in the month prior to PE diagnosis.

### Diagnosis and risk-assessment of PE

The clinical suspicion of PE was confirmed in all subjects by a high probability ventilation-perfusion scan result according to the criteria of the Prospective Investigation of Pulmonary Embolism Diagnosis [14], a lower limb venous compression ultrasonography positive for a proximal deep vein thrombosis in patients with inconclusive or non-diagnostic ventilation-perfusion scans [15], or by contrast-enhanced PE-protocol chest multidetector computed tomography [16].

The risk assessment defined a high-risk PE by the presence of clinically overt right ventricle failure resulting in hemodynamic compromise (shock or persistent arterial hypotension; i.e., a systolic blood pressure <90 mmHg or a pressure drop of >40 mmHg for 15 min); an intermediate-risk PE by the presence of echocardiographic signs of right ventricle dysfunction [17]; and a low-risk PE by exclusion of right ventricle dysfunction and negative myocardial injury markers. These PE risk definitions were applied in both the discovery and the clinical validation studies.

### Plasma proteomic analyses

Detailed information of proteomic techniques, together with the randomization and combination of samples and MS analysis of protein spots, is included in the Minimum Information About Proteomics Experiment (MIAPE) document (Table S1).

Blood samples were obtained in glass vials containing disodium EDTA (Vacutainer K3E 7.2 mg, BD, Franklin Lakes, NJ, USA). Plasma was recovered by centrifugation at 1300×*g* and 4°C for 10 min, and stored at −80°C until assayed. We removed albumin and IgG from plasma applying the albumin and IgG removal kit (GE healthcare, Buckinghamshire, UK) and we dissolved the samples in 1 ml of ice-cold distilled water, and concentrated them by ultrafiltration using a pore-size of 10,000 Da (Amicon Ultra; Millipore, Bedford, MA, USA). Subsequently, we resuspended samples in 200 µl of lysis buffer and applied a second ultrafiltration to obtain a final volume of approximately 70 µl. Finally, we measured protein concentration using the RD-DC Protein Assay (Bio-Rad, Hercules, CA, USA). To evaluate the reproducibility of the extraction, all the samples were separated with 12% SDS-PAGE, 4 µg protein per lane, and silver stained (data not shown) [18].

The techniques used for protein labeling, two-dimensional electrophoresis, 2D-DIGE, image analysis and protein identification have been published earlier [19], [20], [21]. MALDI-TOF/TOF mass spectrometry (MS) analyses were performed in a 4800 Proteomics Analyzer (Applied Biosystems, MDS Sciex, Toronto, Canada) at the Genomics and Proteomics Center, Universidad Complutense, Madrid.

### Experimental design and data analysis

For 2D-DIGE experiments, DeCyder 7.0 (GE Healthcare Bio-Sciences AB, Uppsala, Sweden) differential in gel analysis (DIA) module was used for intra-gel co-detection of samples and internal standard protein spots allowing the detection of an average of 1,707 protein spots (7.85% coefficient of variation [CV]) on each image. Artifactual spots were filtered and removed.

We evaluated the differences in protein abundance in plasma proteins between 6 patients with low-risk PE, and a group of 6 patients that considered, as a whole, 3 subjects with intermediate-risk PE together with 3 subjects with high-risk PE (2 groups, 12 samples, 6 gels). To avoid any possible bias introduced by labeling efficiency, half of the samples from each group were labeled with Cy3 dye and the other half with Cy5 dye. A pool of all the samples labeled with Cy2 served as the internal standard used for normalization of the results. The 18 images from the 6 gels were submitted to DeCyder biological variation analysis module. Biological variation analysis served for inter-gel matching of internal standard and samples across all gels, and for comparative cross-gel statistical analyses of all spots, based on spot volumes, permitting the detection of spots with different abundance between groups. The difference between two samples is reported as a volume ratio. In this step, an average of 1,141 spots was matched on the gels (11.5% CV). The spots volumes of all matched proteins spots were normalized and quantified. Only spots present in 14 out of the 16 gel images were considered. Finally, protein spot matches and data quality were verified manually to exclude artifacts and to avoid false positives. The normalized volume data for each spot obtained from patients and from controls were exported using the XML toolbox and submitted to statistical analysis.

### Haptoglobin assay and genotypes

The circulating concentrations of haptoglobin were assayed by a commercial immunonephelometry method (Dade Behring, Marburg, Germany) calibrated against the international CRM 470 reference material [22] with intra-assay and inter-assay CVs of 2.6% and 6.2%, respectively. Immunonephelometry uses specific antibodies to determine the concentrations of haptoglobin in a suspension. This technique is based on the light-scattering properties of the resulting immune complexes, because the scatter of light and its maximal value increase with the concentration of haptoglobin in the sample.

Genomic DNA was obtained from blood samples using the FujiFilm QuickGene DNA whole blood kit S (FujiFilm Corporation, Tokyo, Japan) and the haptoglobin α–chain polymorphism genotypes were assessed using the polymerase chain reaction-based technique. Two different PCR products were amplified to determine Hp1 and Hp2 alleles using primer pairs A–B and C–D (Sigma-Aldrich), previously described by Koch et al. [23]. Reactions were performed in a 2720 Thermal Cycler (Applied Biosystems) in a final volume of 20 µl containing 2 U of FastStart Taq DNA polymerase (Roche Diagnostics), 50–100 ng of DNA, and 200 µM each dNTP. Reactions with primers A and B required 1.5 mM MgCl_2_ and 5% DMSO and the following amplification protocol: denaturation at 95°C for 5 min, 35 cycles of denaturation at 95°C for 1 min and annealing/extension at 69°C for 3 min, and a final extension at 72°C for 7 min. Reactions with primers C and D required 2 mM MgCl_2_ and the following amplification protocol: denaturation at 94°C for 5 min, 35 cycles of denaturation at 94°C for 1 min, annealing at 69°C for 1 min and extension at 72°C for 1 min, and a final extension at 72°C for 7 min. PCR products were resolved in 0.8% and 1.6% agarose gels, respectively.

### Hemopexin assay

We measured plasma hemopexin concentrations using a commercial ELISA kit (ab108859 Hemopexin Human ELISA kit) following manufacturer's instructions (Abcam plc, Cambridge, UK). The samples from all subjects were assayed in duplicate. The lower limit of detection was 0.07 µg/ml and the mean intra- and inter-assay CVs were 5% and 7.7%, respectively.

### Statistical analysis

We expressed continuous variables as means ± SD and dichotomous variables as counts (percentages) unless otherwise stated. We selected a 1.5-fold difference in protein abundance between groups as the cut-off that excluded experimental variability in comparative proteomic analysis of depleted plasma samples, as determined in our laboratory in previous experiments that used exactly the same methods employed here [19]. After confirming the normal distribution of the variables by the Kolmogorov-Smirnov test, the differences in protein abundance between groups were analyzed by ANOVA. The comparisons of clinical and biochemical variables between the 2 groups of patients with PE included in the discovery study used Mann-Whitney U tests, χ^2^ tests or Fisher's exact tests as appropriate. Differences in serum haptoglobin and hemopexin concentrations among groups in the larger validation study were analyzed by one-way ANOVA followed by the least significant difference *posthoc* test. The accuracy of these protein markers for the diagnosis of high-risk PE was assessed by receiver operating characteristic (ROC) curve analysis. We used SPSS Statistics 17 (SPSS, Chicago, IL) for all statistical analyses. *p*<0.05 was considered statistically significant.

## Results

### Differences in protein abundance between patients with low-risk PE and patients with intermediate or high-risk PE

**Figure 1:**
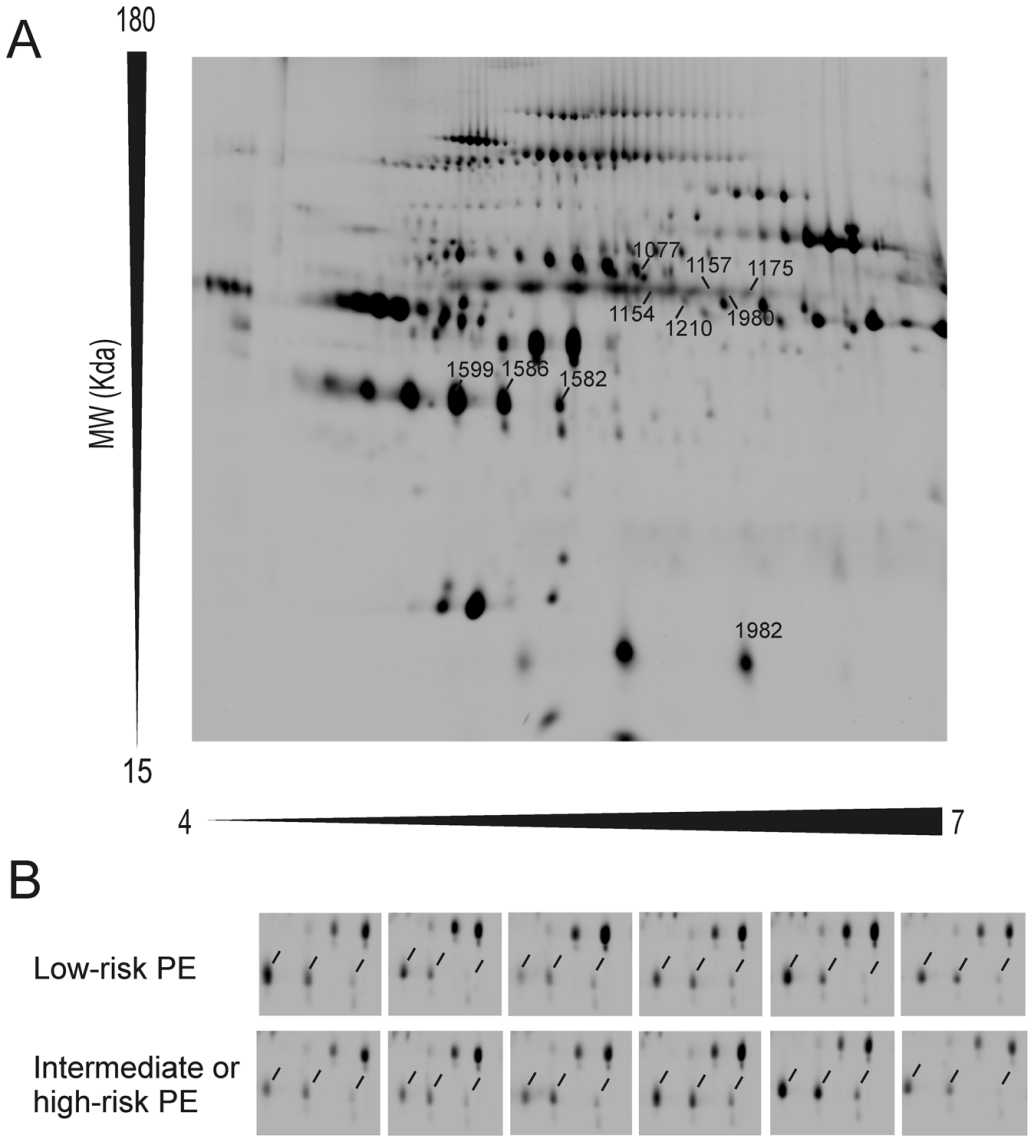
Representative two dimensional-differential in-gel electrophoresis (2D-DIGE) map of depleted plasma proteins including the pick locations of the proteins identified in the study (Panel A). Magnification of the area including the haptoglobin spots (indicated by the line markers) in the 12 gels (Panel B).

The clinical and biochemical characteristics of the patients included in the discovery study are summarized in Table 1. After 2D-DIGE, DeCyder software indicated that the protein abundance of 10 spots reached statistically significant differences, with fold differences above 1.5, among patients with low-risk PE and individuals presenting with intermediate or high-risk PE. Figure 1 shows a representative 2D-DIGE map of plasma protein extracts including the pick locations of the spots identified. After in-gel digestion and MALDI-TOF/TOF analysis, a Mascot database search using the peptide mass fingerprinting spectra allowed the identification of the protein present in 1 spot, whereas the proteins of another 9 spots were identified by tandem mass spectrometry. Detailed information of the identification of these 10 spots is summarized in Table 3, including their accession numbers, theoretical molecular weight (MW) and isoelectric point (pI) values, DeCyder software and identification method parameters.

**Table 3:** Proteins showing different abundances in plasma samples of patients with intermediate and high-risk PE compared with individuals presenting with low-risk PE, detected by nontargeted 2D-DIGE.

		Mascot								Decyder analysis	
			PMF			PMF and MS/MS					
Pos	Protein name	MW/pI	NP	SC	Sco	NP	FP	Sco	Expect	*P* value	Average ratio
1077	Hemopexin	52/6.5	16	34	117/38					0.049	−1.58
1154	Ig alpha-1 chain C region	38/6.1				12	2	149	<0.001	0.025	1.62
1157	Ig alpha-1 chain C region	38/6.1				9	1	90	<0.001	0.007	1.92
1175	Ig alpha-1 chain C region	38/6.1				13	2	248	<0.001	0.022	2.22
1980	Ig alpha-1 chain C region	38/6.1				14	5	346	<0.001	0.004	2.00
1582	Haptoglobin	45/6.1				6	2	57	<0.001	0.044	−1.93
1586	Haptoglobin	45/6.1				11	1	127	<0.001	0.024	−2.04
1599	Haptoglobin	45/6.1				18	2	256	<0.001	0.034	−1.56
1210	Alpha-2-macroglobulin	122/5.4				16	3	193	<0.001	0.021	1.57

Pos: Position numbers correspond to the position of the proteins in the gel image (Figure 1).

MW/pI: Theoretical molecular weight (MW) in kDa and theoretical pI.

PMF: peptide mass fingerprinting.

NP: Number of peptide mass values matched from MASCOT PMF.

SC: Amino acid sequence coverage for the identified proteins.

Sco: MASCOT MS protein score, obtained from MALDI-TOF/TOF spectra from the top hit and the second one. Larger differences indicate a better result.

FP: Number of fragmented peptide masses by tandem mass spectrometry.

Expect: Quality of an individual match. It is the number of times in the search we could expect to get a match with this score or higher by chance.

The 10 spots showing differences in abundance corresponded to only 4 proteins, because 2 of the proteins identified were present in more than one spot suggesting post-translational modification or proteolysis. 2D-DIGE analysis showed that four protein species corresponding with Ig α1-chain C region and one protein species of α2-macroglobulin were increased in the plasma samples from patients with intermediate or high-risk PE. On the other hand, three protein species corresponding to haptoglobin and one protein species of hemopexin were decreased compared with patients with acute low-risk PE.

### Validation of candidate markers in the clinical validation population

We then proceeded to validate in a separate clinical population the two candidate biomarkers (hemopexin and haptoglobin) more likely to play a role in PE according to our current knowledge about the pathophysiology of this disorder. This population consisted of 52 adults with intermediate (n = 47) or high-risk (n = 5) PE, who were compared with 52 patients with low-risk PE, whose clinical and biochemical characteristics are summarized in Table 2.

**Figure 2:**
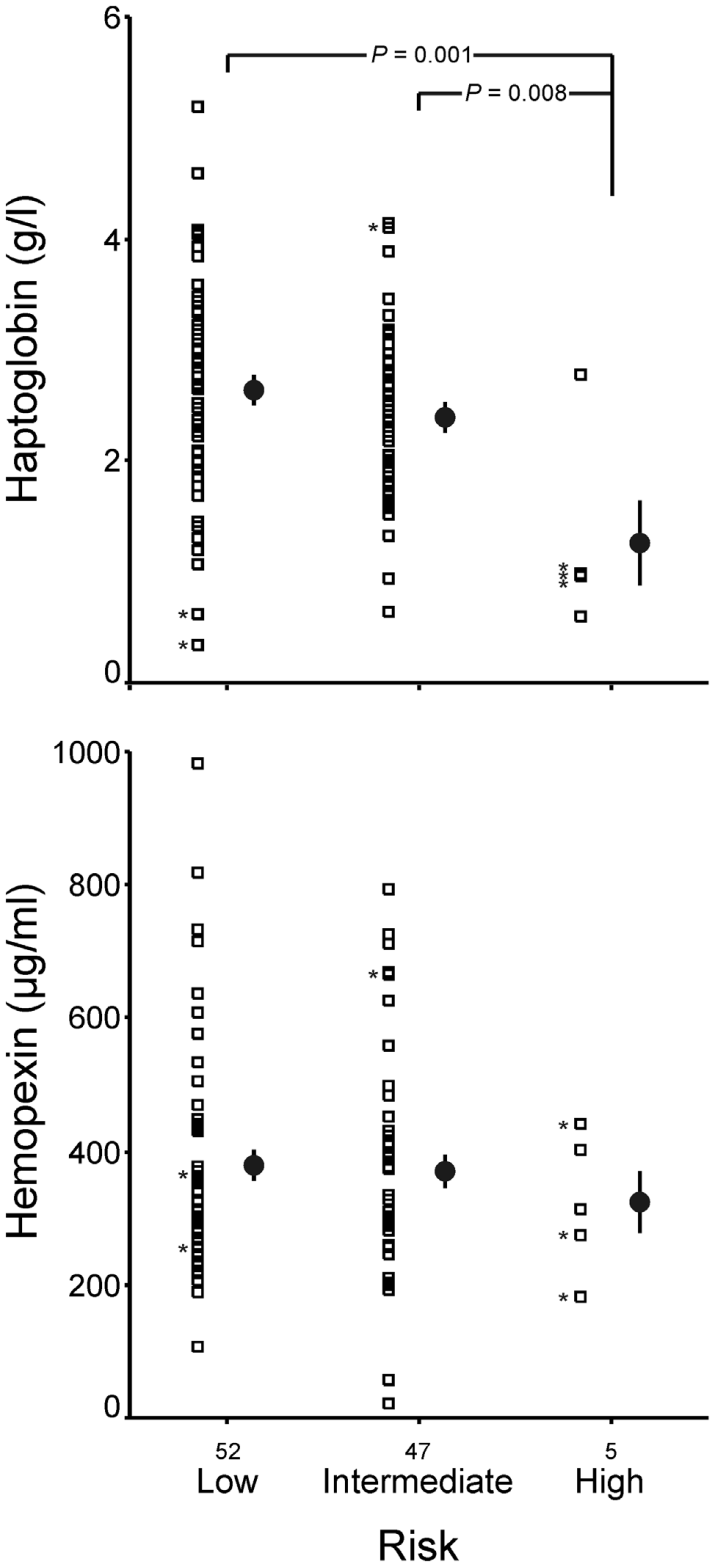
Serum haptoglobin and hemopexin concentrations in the diagnostic validation series consisting of 52 patients with low-risk pulmonary embolism (PE), 47 patients with intermediate PE and 5 patients with high-risk PE. Bars are means and 95% confidence intervals. Asterisk represents patients who died of PE. Data were submitted to one-way ANOVA followed by the least-significant difference *posthoc* test. Serum haptoglobin concentrations were determined by immunonephelometry and hemopexin levels were measured by ELISA.

Confirming proteomic results, patients with intermediate or high-risk PE exhibited a significant decrease in serum concentrations of haptoglobin compared with patients with low-risk PE (2.3±0.8 vs 2.6±1.0 g/l respectively, *P* = 0.045), and such a decrease was especially marked in patients with high-risk PE (Figure 2). On the contrary, hemopexin concentrations as determined by ELISA did not show statistically significant differences between the groups of patients with PE of different severity, either when comparing the 52 patients with intermediate or high-risk PE with the 52 patients with low-risk PE (366±163 vs 379±170 µg/ml respectively, *P* = 0.684), or when comparing low-risk, intermediate-risk and high-risk PE patients separately (Figure 2).

Because Hp2 alleles of the haptoglobin α–chain polymorphism reduce serum concentrations and anti-oxidant properties and increase the pro-inflammatory actions of this acute-phase protein in a gene-dosage fashion [24], we analyzed the distribution of this polymorphism in the validation study to find out if the differences observed in serum haptoglobin concentrations in patients with high-risk PE were dependent on a genetic predisposition.

In our series, haptoglobin α–chain genotypes were distributed according to Hardy-Weinberg equilibrium in all the groups of patients (data not shown). As expected from previous studies [24], subjects homozygous for Hp2 alleles as a whole showed decreased circulating levels of haptoglobin compared with subjects carrying Hp1 alleles (Hp1/1: 2.6±0.6 g/l; Hp1/2: 2.7±0.9 g/l; Hp2/2: 2.1±0.9 g/l, P = 0.003). The distribution of haptoglobin genotypes was not different between the low-risk PE patients and those presenting with intermediate or high-risk PE (low-risk PE: Hp1/1, n = 7, Hp1/2, n = 22, Hp2/2, n = 23; intermediate or high-risk PE: Hp1/1, n = 8, Hp1/2, n = 25, Hp2/2, n = 19; χ^2^ = 0.639, *P* = 0.726, the intermediate and high-risk categories had to be combined into a category to meet the χ^2^ requirement that less than 20% of the expected frequencies are less than 5).

**Figure 3:**
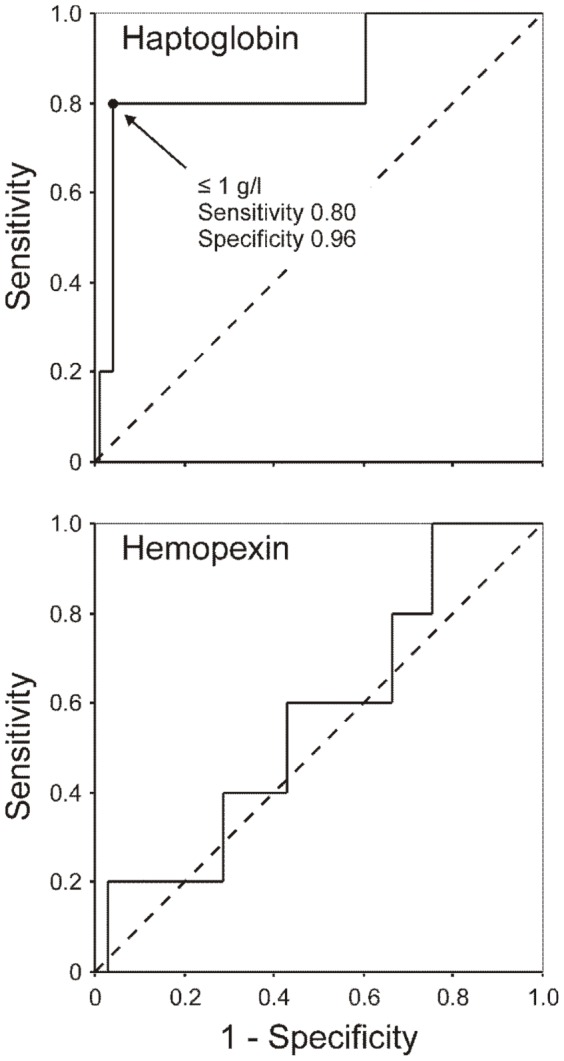
Receiver operating characteristic curve analysis of serum haptoglobin and hemopexin concentrations for the diagnosis of high-risk pulmonary embolism. The areas under the ROC curves were 0.853 (95% confidence interval 0.648−1.057, *P* = 0.008) for haptoglobin, and 0.567 (95% confidence interval 0.329−0.806, *P* = 0.613) for hemopexin.

We used separate ROC curve analyses to assess the diagnostic accuracy of circulating haptoglobin and hemopexin as biomarkers of high-risk PE (Figure 3). Circulating haptoglobin showed a 0.853 area under the ROC curve (95% confidence interval 0.648−1.057, *P* = 0.008) for the detection of high-risk PE in our series, whereas circulating hemopexin was not useful for this purpose showing a 0.567 area under the ROC curve (95% confidence interval 0.329−0.806, *P* = 0.613). A circulating haptoglobin value equal or below 1 g/l showed a 80% sensitivity and a 96% specificity for the diagnosis of high-risk PE.

Finally, 6 of the 104 patients included in the validation sample (5.8%; 95% CI of the proportion: 2.4%−12.6%) died from PE within thirty days of presentation. For 2 patients in the validation cohort who died, reclassification was more accurate when haptoglobin was combined with the presence of high-risk PE, resulting in a 33.3% increase in nonsurvivors correctly identified as being at high risk. The net improvement in reclassification was estimated at 0.32 when considering serum haptoglobin values.

## Discussion and Conclusions

Our present nontargeted proteomic discovery study has identified a panel of protein markers whose serum concentrations are significantly altered according to the severity of PE. The functions of these proteins points to novel mechanistic pathways indicating the involvement of iron metabolism and complement pathways, and of the coagulation cascade in the mechanisms that determine the severity of PE. We have validated these results in a second clinical population of patients with PE identifying reduced haptoglobin concentrations as a serum protein biomarker that may be used in combination with clinical and echocardiographic data with the aim to identify patients at high-risk of PE-related complications.

The biomarker panel comprises positive acute-phase proteins such as α2-macroglobulin and Ig α_1_-chain C region (that were more abundant) and haptoglobin and hemopexin (that were less abundant) in patients with high-risk compared with less severe PE patients. The acute-phase response is an innate body defense observed during infection, physical trauma, malignancy, and tissue damage that aims to minimize on-going tissue damage by isolating and destroying infective agents while activating repair processes. The innate immune response involves the recruitment and activation of macrophages and leukocytes that release inflammatory cytokines on recognition of a pathogen-associated molecular pattern. These cytokines travel through the bloodstream and stimulate hepatocytes in the liver to synthesize and secrete acute-phase proteins.

The acute-phase proteins identified here in association with the severity of PE are predominantly liver-synthesized proteins that have an important antiinflammatory activity through inhibition of oxidative stress and iron sequestration resulting in antimicrobial activity. As such, they may function to modulate the systemic inflammatory response to inflammation [25] and be involved in tissue repair through fibrosis and angiogenesis [26], [27].

In addition, both haptoglobin and hemopexin are involved in iron metabolism. The inhibition of heme release from hemoglobin by haptoglobin and sequestration of heme by hemopexin suppress hemoglobin-mediated oxidative stress, attenuate endothelial cytotoxicity, and protect cells from heme toxicity [24], [28]. In addition, hemoglobin and its derivative heme are often released into tissue compartments where there is inflammation, in the presence of degrading blood, and hemoglobin synergizes with multiple toll-like receptor agonists to induce release of high levels of tumor necrosis factor and interleukin-6 from macrophages, an effect that is attenuated by hemopexin [29]. Hemopexin also down-regulates lipopolysaccharide-induced proinflammatory cytokine release from macrophages [30].

Of the 4 proteins identified by the nontargeted proteomic study we were able to confirm, in a much larger validation study, that serum haptoglobin concentrations are actually decreased in the most severe forms of PE, in conceptual agreement with previous animal and human studies on the issue [31], [32], [33]. Severe PE causes turbulent flow across the tricuspid and pulmonic valves and in the pulmonary tree and may rupture red cells in, or immediately proximal to, or within, the pulmonary vascular tree [31]. Hemolysis then results in a transient increase in unbound hemoglobin and free heme immediately proximal to the lung. This timing and location of this shear effect requires release of an only a small amount of free hemoglobin to have a profound effect on pulmonary vascular tone. Upon its rupture, the erythrocyte releases tetrameric (α2β2) hemoglobin, which can avidly bind nitric oxide, but must first dissociate into αβ dimers before haptoglobin can bind and inactivate this nitric oxide scavenging effect [34].

The decrease in serum haptoglobin found in patients high-risk PE could result from at least two hypothetical mechanisms. On the one hand, because haptoglobin's protective role against intravascular hemolysis, possibility existed that subjects carrying Hp2 alleles of the haptoglobin α–chain polymorphism, by having lower serum haptoglobin levels, would be at risk for more severe consequences of PE (i.e. their defense against intravascular hemolysis would have been less effective). Our present results, showing no association of Hp alleles with the severity of PE, weight heavily against this possibility. On the other hand, consumption of haptoglobin in order to prevent the liberation of heme from the intravascular hemoglobin liberated in severe PE during intravascular hemolysis may explain the decrease in haptoglobin concentrations observed in our high-risk PE patients [24]. Other disorders that may decrease haptoglobin concentration such as malnutrition or severe hepatic disease [24] were not present in the patients studied here.

A possible limitation of our study is that the diagnostic utility of plasma haptoglobin as a biomarker of hemolysis in patients with PE might be confounded by a positive acute phase reactant effect, resulting in spuriously elevated haptoglobin concentrations, probably as a result of the underlying inflammatory and thrombophilic factors that caused the patient's venous thrombosis [24]. However, the significant results from the validation cohort, showing reduced serum haptoglobin levels in patients with severe PE despite the possible interference of a positive acute phase response, provided evidence of the robustness of the findings and further strengthened the soundness of the abovementioned hypothesis.

Even though another potential limitation of the study is the small number of patients in the high risk stratification group, it is noteworthy that ROC curve analysis indicated that reduced serum haptoglobin concentrations show a very good diagnostic accuracy for high-risk PE – a single haptoglobin determination has an 85% chance of correctly identifying patients with high-risk PE – and that using a ≤1 g/l cut-off value, serum haptoglobin concentration has 80% sensitivity and 96% specificity for the detection of the most severe forms of PE in a clinical setting. These promising results require future confirmation in larger series of unselected patients with PE.

In conclusion, this report shows that circulating haptoglobin is decreased in patients with severe PE. The decrease in serum haptoglobin may arise from a protective response against intravascular hemolysis, further supporting current hypotheses that suggest intravascular hemolysis as a contributor to pulmonary hypertension in patients with severe acute PE. Finally, reduced serum haptoglobin concentration should be considered a promising protein marker of the most severe forms of PE.

## Supporting Information

Table S1 Minimun Information About a Proteomics Experiment (MIAPE) report file generated by MIAPE generator tool (http://www.proteored.org/).
(DOC)
