# Supplementary material for: Identification of Reduced Circulating Haptoglobin Concentration as a Biomarker of the Severity of Pulmonary Embolism: A Nontargeted Proteomic Study
Source: PLoS One. 2014 Jun 30;9(6):e100902. doi: 10.1371/journal.pone.0100902 (PMC4076207; doi:10.1371/journal.pone.0100902)
Supplement: Table S1 — Minimun Information About a Proteomics Experiment (MIAPE) report file generated by MIAPE generator tool (http://www.proteored.org/). (DOC) [file pone.0100902.s001.doc]

Supplemental Data Table 1: MIAPE DOCUMENT

| | **Gel Electrophoresis MIAPE document** | | --- | | Based on the original guidelines: [MIAPE GE v1.4](http://estrellapolar.cnb.csic.es/proteored/Biblioteca/Ficheros/367483048_MIAPE_GE_1_4.pdf) | | |
| --- | --- | --- | --- |
| |  | | --- |  | | 1. General features | | --- | | |  | | --- | | **Date stamp:** | | 1/13/2013 | | **Name:** | | Proteomics of pulmonary embolism | | **Last update:** | | 2/12/2013 9:58:10 AM | |  | | **Electrophoresis type:** | | Difference gel electrophoresis | |  | | **2. Sample** | | **Name:** | | Differential protein abundance in plasma proteins | | **Description:** | | Plasma of 12 patients with acute symptomatic pulmonary embolism (PE):   6 patients with nonmassive PE  6 matched patients with submassive (n = 3) or massive (n = 3) PE   Experimental Design:   GEL Cy3 Cy5 Cy2   1 nonmassive PE 1 massive PE 1 Pool   2 nonmassive PE 2 massive PE 2 Pool   3 nonmassive PE 3 massive PE 3 Pool   4 massive PE 4 nonmassive PE 4 Pool   5 massive PE 5 nonmassive PE 5 Pool   6 massive PE 6 nonmassive PE 6 Pool | |  | | **3. Protocol** | | **Name:** | | DIGE_Protocol | | **3.1 First dimension (1/2)** | | **Name:** | | First Dimension | | **Ordinal number for this dimension: 1** | |  | | **Separation method employed: IEF** | |  | | **3.1.1 Loading buffer** | | **Name:** | | Rehydration buffer 4-7 Urea/Thiourea | | **Type:** | | Rehydration buffer | | **Comments:** | | Rehydration method: passive  Rehydration time: 12h | |  | | **3.1.1.1 Buffer components** | | Urea - 7M | | Thiourea - 2M | | CHAPS - 2% | | IPG buffer 4-7 - 2% | | Bromophenol blue - 0.01% | | DTE - 65mM | |  | | **3.1.2 Gel matrix** | | **Name:** | | Immobilized pH gradient gel | | **Type of the gel matrix:** | | Polyacrylamide | | **Gel manufacture:** | | Immobiline DryStrip, 17-6003-77, GE Healthcare | | **Physical dimensions:** | | x: 24 | | y: 0.3 | | z: 0.1 | | Unit: centimeter | | **PH range and distribution:** | | Lower limit: 4 | | Upper limit: 7 | | Range type: linear distribution | |  | | **3.1.2.4 Sample application** | | **Name:** | | Cup loading | | **Comments:** | | Every sample is loaded upon one strip by using *cup loading*  Sample loaded: 150μg (1 μg/ul) | |  | | **3.1.3 Electrophoresis Protocol** | | **Name:** | | IEF 24 cm, 4-7, *cup loading* | | **Electrophoresis conditions:** | | 1- Step 120 V 1 h  2- Step 1000 V 1 h  3- Step 3500 V 22 h  4- Step 5000 V 30 min  5- Step 50V 8 h  6- Step 500 V 8 h   100uA/Strip | |  | | **3.1 Second dimension** | | **Name:** | | Second Dimension | | **Ordinal number for this dimension: 2** | |  | | **Separation method employed:** SDS-PAGE | |  | | **3.1.2 Gel matrix** | | **Name:** | | Polyacrylamide gel | | **Type of the gel matrix:** | | Denaturing gel 10% | | **Physical dimensions:** | | x: 26 | | y: 22 | | z: 0.1 | | Unit: centimeter | | **MW range and distribution:** | | Lower limit: 15.0 | | Upper limit: 200.0 | | Unit: kilodalton | | Range type: logarithmic distribution | | **Acrylamide concentration:** | | 10 | | **Acrylamide : Bisacrylamide ratio:** | | 30 : 0.8 | |  | | **3.1.2.1 Polymerization buffers (not in HUPO-PSI MIAPE documents)** | | **Name:** | | 10% Poly-Acrylamide buffer | | **Type:** | | Resolving buffer | |  | | **3.1.2.1.1 Buffer components (not in HUPO-PSI MIAPE documents)** | | Acrilamide 30% | | PDA 2% | | Tris-HCl 1.5M - pH 8.8 | | SDS 10% | | MiliQ water | | Amonium persulphate -10% | | TEMED | |  | | **3.1.2.2.1 Buffer components** | | Tris Base - 25mM | | Glycine - 192mM | | SDS - 0.2% | | Agarose - 0.5% | | Bromophenol Blue - 0.002% | |  | | **3.1.3 Electrophoresis Protocol** | | **Name:** | | SDS-PAGE electrophoresis | | **Electrophoresis conditions:** | | 2 W /gel 16 hrs | |  | | **3.1.3.1 Running Buffer (1/2)** | | **Name:** | | Lower: SDS-PAGE electrophoresis Buffer 1X | | **Type:** | | Running Buffer | |  | | **3.1.3.1.1 Buffer components** | | Tris Base - 25mM | | Glycine - 192mM | | SDS - 0.1% | |  | | **3.1.3.1 Running Buffer (2/2)** | | **Name:** | | Upper SDS Electrophoresis Buffer 2X | |  | | **3.1.3.1.1 Buffer components** | | Tris Base - 50mM | | Glycine - 384mM | | SDS - 0.2% | |  | | **3.1.3.2 Additional Buffer** | | **Name:** | | Agarose buffer 0.5% | | **Type:** | | Agarose sealing solution | |  | | **3.1.3.2.1 Buffer components** | | Tris Base - 25mM | | Glycine - 192mM | | SDS - 0.2% | | Agarose - 0.5% | | Bromophenol Blue - 0.002% | |  | | **3.2 Inter-dimension Step** | | **Step name:** | | Equilibration | | **Protocol:** | | Step 1 (reduction): 15 min at room temperature with shaking  Step 2 (alkylation): 15 min at room temperature with shaking | |  | | **3.2.1 Inter-dimension Buffer (1/2)** | | **Name:** Reduction buffer DTE 2% | |  | | **3.2.1.1 Buffer components** | | Tris ph 6.8 - 100mM | | Urea - 6M | | Glicerol - 30% | | SDS - 2% | | Bromophenol Blue - 0.002% | | DTE - 2% | |  | | **3.2.1 Inter-dimension Buffer (2/2)** | | **Name:** Alkylation buffer Iodoacetamide 2.5% | |  | | **3.2.1.1 Buffer components** | | Tris ph 8.0 - 100mM | | Urea - 6M | | Glicerol - 30% | | SDS - 2% | | Bromophenol Blue - 0.002% | | Iodoacetamide - 2.5% | |  | | **4. Direct detection (1/3)** | | **Name of direct detection process:** | | Direct Detection using Typhoon | | **Protocol:** | | DIGE fluorescence was detected by using a Typhoon 9400 scanner | |  | | **4.1 Direct detection agents** | | Cy2 | | Cy3 | | Cy5 | |  | | **4. Direct detection (2/3)** | | **Name of direct detection process:** | | Coomassie staining | | **Protocol:** | | 1- Fixation in 50% EtOH, 2% phosphoric acid for 3h O/N 2- Rinse 3x10 min with Mili-Q H_2_O  3- Stain. 85g amonium sulphate, 15ml phosphoric acid 330ml Mili-Q H_2_O and 165 ml methanol. Shake for 1 hour 4- Add 330mg of Coomassie Blue G-250 resuspended in 5ml Methanol O/N  5- Shake in water to get proper contrast | |  | | **4.1 Direct detection agents** | | Coomassie blue | |  | | **4. Direct detection (3/3)** | | **Name of direct detection process:** | | Silver Staining | | **Protocol:** | | 1- 50% methanol 10% acetic acid Fix 30 min 2- 5% methanol Incubate 15 min 3- milli-Q H_2_O 3 x 5 min 4- sodiumthiosulphate (0.2g/L)120 sec 5- milli-Q H_2_O 3 x 30 sec 6- Silver nitrate (2g/L) 25 min 7- milli-Q H_2_O 3 x 60 sec 8- Sodium carbonate (30g/L) 10 min 9- Na_2_-EDTA (14g/L) 10 min | |  | | **6. Image Acquisition** | | **Name:** | | Adquisition of DIGE images. 6 gels. | | **Protocol:** | | All gel images were acquired with the following settings:  Emission filter for Cy2: 520 BP40  Emission filter for Cy3: 580 BP30  Emission filter for Cy5: 670 BP30  Image resolution: 100 dots/cm | |  | | **6.1 Equipment** | | **Name:** | | Typhoon Scanner | | **Type of equipment:** | | Laser scanner | | **Equipment specific parameters:** | | Lasers:  488 nm for Cy2  532 nm for Cy3  633 nm for Cy5  Emission filters:  520 BP40 for Cy2  670 BP30 for Cy5  580 BP30 for Cy3 | |  | | **6.2 Image Acquisition Software** | | **Name:** | | Typhoon Scanner Control Software | | **Version:** | | v3.0 | | **Vendor:** | | GE | | **Description:** | | Typhoon Scanner Software v3.0 and ImageQuant TL v2003.02 | |  | | | | --- | --- | --- | --- | --- | --- | --- | --- | --- | --- | --- | --- | --- | --- | --- | --- | --- | --- | --- | --- | --- | --- | --- | --- | --- | --- | --- | --- | --- | --- | --- | --- | --- | --- | --- | --- | --- | --- | --- | --- | --- | --- | --- | --- | --- | --- | --- | --- | --- | --- | --- | --- | --- | --- | --- | --- | --- | --- | --- | --- | --- | --- | --- | --- | --- | --- | --- | --- | --- | --- | --- | --- | --- | --- | --- | --- | --- | --- | --- | --- | --- | --- | --- | --- | --- | --- | --- | --- | --- | --- | --- | --- | --- | --- | --- | --- | --- | --- | --- | --- | --- | --- | --- | --- | --- | --- | --- | --- | --- | --- | --- | --- | --- | --- | --- | --- | --- | --- | --- | --- | --- | --- | --- | --- | --- | --- | --- | --- | --- | --- | --- | --- | --- | --- | --- | --- | --- | --- | --- | --- | --- | --- | --- | --- | --- | --- | --- | --- | --- | --- | --- | --- | --- | --- | --- | --- | --- | --- | --- | --- | --- | --- | --- | --- | --- | --- | --- | --- | --- | --- | --- | --- | --- | --- | --- | --- | --- | --- | --- | --- | --- | --- | --- | --- | --- | --- | --- | --- | --- | --- | --- | --- | --- | --- | --- | --- | --- | --- | --- | --- | --- | --- | --- | --- | --- | --- | --- | --- | --- | --- | --- | --- | --- | --- | --- | --- | --- | --- | --- | --- | --- | --- | --- | --- | --- | --- | --- | --- | --- | --- | --- | --- | --- | --- | --- | --- | --- | --- | --- | --- | --- | |  |

| | **Gel Electrophoresis Informatics MIAPE document** | | --- | | Based on the original guidelines: [MIAPE GI v1.0](http://estrellapolar.cnb.csic.es/proteored/Biblioteca/Ficheros/182427582_miape-gi-v1.doc) | | | |  | | | |
| --- | --- | --- | --- | --- | --- | --- | --- | --- |
| |  | | --- |  | | | **1. General features** | | --- | | **Name:** | | MIAPE GI DIGE | | **1.2 Electrophoresis type:** | | Difference gel electrophoresis | |  | | **1.5 Image analysis software** | | DeCyder - Version: v7.0 | | **Vendor:** | | Ge HEalthcare | | **Description:** | | Decyder is image analysis software that significantly increases throughput by accurately addressing measurement of protein differences with statistical confidence. DeCyder Differential Analysis Software automatically detects, matches and analyzes protein spots in multiplexed fluorescent images, and is able to give routine detection of < 10% differences with > 95 % confidence. Statistical analysis is carried out on each and every difference. | |  | | **2. Gel analysis design** | | **2.1 Type:** | | The analysis design with respect to the assignment of images to groups were direct, it means the groups were defined by the investigator. | | **2.2 Replicates:** | | Six biological replicates of each group. | | **2.3 Groups:** | | Two groups: 6 patients with acute nonmassive PE, and 6 matched patients with submassive (n = 3) or massive (n = 3) PE. | | **2.4 Standards:** | | Internal standard: a pool of all the samples labeled with Cy2. | |  | | **3. Image preparation** | |  | | **3.2 Image preparation step** | | **Name:** | | Croping step | | **Type:** | | Regular crooping | | **5. Data extraction process** | | **Name:** | | Data extraction for DIGE | |  | | **5.1 Feature detection** | | **Algorithm name/version:** | | Decyder Software - Version: Batch processor | | **Feature editing:** | | Stimated number of spots:10000  Spot exclusion filter: volume 30000 | | **Parameters:** | | The Batch Processor links both the DeCyder Differential Analysis Software DIA and BVA modules to perform all stages of the 2-D DIGE analysis process.  Once the Batch Processor has been set up, the gels are processed sequentially without user involvement. | |  | | **5.2 Matching** | | **Algorithm name/version:** | | Spot matching algorithm in DIGE | | **Landmarks:** | | Yes | | **Match editing:** | | Yes | | **Parameters:** | | Gel images were scanned on Typhoon™ scanner, and added to the DeCyder DIA workspace via Batch processor. Spots on the gel are then detected automatically and spot maps created for 3 images/gel, all having the same spots or spot number.  This module performs spot co-detection and spot quantification by normalization and ratio calculation. | | **5.3 Feature quantitation** | | **Algorithm name/version:** | | Feature quantitation in DIGE | | **Type:** | | Spot Volume, not Normalize | |  | | **5.3.2 Background subtraction** | | Not performed | |  | | **5.3.3 Normalization** | | Normalization | | **Parameters:** | | DeCyder BVA analyzes multiple sets of spot maps detected in DIGE images by the DeCyder DIA Differential in-gel Analysis software. Spots are automatically matched between the different spot maps and statistical analysis can easily be performed using built-in functions. | |  | | **6. Data analysis** | | **Input data:** | | Logarithm of the standardized protein abundances of the spot | | **Analysis intent:** | | We used a 1.5-fold change in the protein abundance of the depleted plasma samples as the threshold value that excluded an influence of technical variability | | | --- | --- | --- | --- | --- | --- | --- | --- | --- | --- | --- | --- | --- | --- | --- | --- | --- | --- | --- | --- | --- | --- | --- | --- | --- | --- | --- | --- | --- | --- | --- | --- | --- | --- | --- | --- | --- | --- | --- | --- | --- | --- | --- | --- | --- | --- | --- | --- | --- | --- | --- | --- | --- | --- | --- | --- | --- | --- | --- | --- | --- | --- | --- | --- | --- | --- | --- | --- | --- | --- | --- | | | --- | --- | --- | --- | --- | --- | --- | --- | --- | --- | --- | --- | --- | --- | --- | --- | --- | --- | --- | --- | --- | --- | --- | --- | --- | --- | --- | --- | --- | --- | --- | --- | --- | --- | --- | --- | --- | --- | --- | --- | --- | --- | --- | --- | --- | --- | --- | --- | --- | --- | --- | --- | --- | --- | --- | --- | --- | --- | --- | --- | --- | --- | --- | --- | --- | --- | --- | --- | --- | --- | --- | --- | |  |  | |  | | |
| | **Mass Spectrometry MIAPE document** | | --- | | Based on the latest version of the guidelines: [MIAPE MS v2.97](http://estrellapolar.cnb.csic.es/proteored/Biblioteca/Ficheros/106159725_MIAPE_MS_2_97.doc) | | | | | | | |
| |  | | --- |  | | | **1. General features** | | --- | | **Document name:** | | MIAPE MS (MALDI-TOF-TOF) | |  | | **Responsible person** | | **Name:** | | Lola Gutiérrez Blázquez | | **Telephone:** | | 91.394.16.13 | | **Email:** | | cai.proteomica@pas.ucm.es | | **Address:** | | Pza Ramón y Cajal, s/n | | **Locality:** | | Madrid | | **Country:** | | Spain | | **CP:** | | 28040 | | **Department:** | | Proteomics Facility | | **Institution:** | | Centro de Genómica y Proteómica  Unidad de Proteómica  Facultad de Farmacia - UCM | | **Resolution for all MS modes for which dada are presented:** | | 2.9 (FWHM) | |  | | **Estimated mass accuracy for all MS levels for which data are presented:** | | 50 ppm (peptide tolerance) | |  | | **Trypsin digestion and extraction** | | Proteins selected for analysis were in-gel reduced, alkylated and digested with trypsin. Spots were washed twice with water, shrunk 15 min with 100% acetonitrile and dried in a vacuum evaporator (Savant SpeedVac) for 30 min. Then, the samples were reduced with 10 mM dithioerytritol in 25 mM ammonium bicarbonate for 30 min at 56 °C and subsequently alkylated with 55 mM iodoacetamide in 25 mM ammonium bicarbonate for 20 min in dark. Finally, samples were digested with 12.5 ng/µl sequencing-grade trypsin (Roche Molecular Biochemicals) in 25 mM ammonium bicarbonate (pH 8.5) for at least 6 h at 37 °C. | | **Protein and matrix spotting** | | After digestion, 1 ul was spotted onto a MALDI target plate and allowed to air-dry at room temperature. Then, 0.4 ul of a 3mg/ml of α -cyano-4-hydroxy-transcinnamic acid matrix (Sigma) in 50% acetonitrile were added to the dried peptide digest spots and allowed again to air-dry at room temperature. | |  | | **1.1 Instrument** | | **Name:** | | 4800 Plus MALDI TOF-TOF Analyzer | | **Vendor:** | | Applied Biosystems instrument model | | **Any significant deviation from the manufacturer's speccifications for the mass spectrometer:** | | 4993 | |  | | **Instrument configuration** | |  | | **2. Ion Sources** | |  | | **2.2 MALDI** | | **Name:** | | 4800 plus MALDI-TOF-TOF Analyzer, AB | | **Plate composition (or type):** | | 384 Opti-TOF™ (123x81mm) REV A stainless steel | | **Matrix composition:** | | cyano-4-hydroxy-trans-cinnamic acid matrix (Sigma) | | **PSD (or LID/ISD) summary:** | | Collission Induced Disociation (CID)  Selection of ion precursor (Timed Ion Selector)  Reduction of energy of ions before collision cell (Deceleration Stack 6.30 Kv). In the collision cell the ion precursors are fragmented for collision with atmosferic gas and then are reaccelerated in source 2 with variable voltage and ground to get fine-tune spectrum.  Collision Cell 7.00 Kv | | **Operation with or without delayed extraction:** | | Delayed | | **Laser type:** | | Nitrogen Laser Diode-pumped Nd:YAG | | **Wavelength in nanometers:** | | 355 | | **Other laser related parameters:** | | Pulse rate up to 200 Hz with < 500 psec duration/pulse | |  | | **4. Spectrum and peak list generation and annotation** | |  | | **4.1 Data acquisition** | | **Software name:** | | AB SCIEX TOF/TOF Series Explorer Software | | **Version:** | | V3.5.28193 (build 1011) | | **Vendor:** | | Applied Biosystems | | **Location of 'parameters file':** | | [\MS Reflector Positivo (MS Acquisition Method); \MSMS 1kV Positivo (MSMS Acquisition Method)](http://estrellapolar.cnb.csic.es/MS Reflector Positivo (MS Acquisition Method); /MSMS 1kV Positivo (MSMS Acquisition Method)) | | **Used for:** | | 4000 Series Explorer 3.5.2 (Spectra Acquisition and Processing Software ; GPS Explorer 3.6 (Automatic Protein Database Searches); Data Explorer 4.9.6 (Spectra Reprocessing Software) | |  | | **4.2 Data analysis** | | **Name:** | | 4800 Peak list generation | |  | | 4.3 Resulting data | | | **File name** | | --- | | 4800 Spectra Description | | | | --- | --- | --- | --- | --- | --- | --- | --- | --- | --- | --- | --- | --- | --- | --- | --- | --- | --- | --- | --- | --- | --- | --- | --- | --- | --- | --- | --- | --- | --- | --- | --- | --- | --- | --- | --- | --- | --- | --- | --- | --- | --- | --- | --- | --- | --- | --- | --- | --- | --- | --- | --- | --- | --- | --- | --- | --- | --- | --- | --- | --- | --- | --- | --- | --- | --- | --- | --- | --- | --- | --- | --- | --- | --- | --- | --- | --- | --- | --- | --- | --- | --- | --- | --- | --- | --- | --- | | | --- | --- | --- | --- | --- | --- | --- | --- | --- | --- | --- | --- | --- | --- | --- | --- | --- | --- | --- | --- | --- | --- | --- | --- | --- | --- | --- | --- | --- | --- | --- | --- | --- | --- | --- | --- | --- | --- | --- | --- | --- | --- | --- | --- | --- | --- | --- | --- | --- | --- | --- | --- | --- | --- | --- | --- | --- | --- | --- | --- | --- | --- | --- | --- | --- | --- | --- | --- | --- | --- | --- | --- | --- | --- | --- | --- | --- | --- | --- | --- | --- | --- | --- | --- | --- | --- | --- | --- | | | | | |  |  |

| | **Mass Spectrometry Informatics MIAPE document** | | --- | | Based on the latest draft guidelines: MIAPE MSI v1.2 | | | |
| --- | --- | --- | --- | --- |
| |  | | --- |  | | | **1. General features** | | --- | | **Name:** | | MIAPE MS MALDI Informatics | |  | | **1.1 Software package(s) (1/3)** | | GPS Explorer - Version: 3.6 | | **Vendor:** | | Applied Biosystems | | **Used for:** | | Searches Interface. It allows to load the MALDI peak lists and submit the searches against the protein databases. | |  | | **1.1 Software package(s) (2/3)** | | Data Explorer - Version: 3.6 | | **Vendor:** | | Applied Biosystems | | **Used for:** | | Manual spectra processing | |  | | **1.1 Software package(s) (3/3)** | | Mascot - Version: 2.1 | | **Vendor:** | | Matrixscience | | **Used for:** | | Protein identification | | **Availability:** | | <http://www.matrixscience.com/search_form_select.html> | |  | | **2. Input data and parameters** | |  | | **2.1 Input data sets** | | **Name:** | | Peptide Mass Fingerprinting MALDI-TOF/TOF | |  | | **2.1.1 Input Data** | | **Name:** | | Peptide Mass Fingerprinting MALDI-TOF/TOF | | **Description of MS data:** | | Applied Biosystems T2D raw data | | **Type of MS data:** | | AB SCIEX TOF/TOF T2D file | |  | | **2.2 Input parameters** | | **Parameters applied in software (from section 1.1):** | | Mascot | | **Name:** | | Mascot - Search Parameters - Trypsin | | **Search type:** | | combined pmf + ms-ms search | | **Taxonomical restrictions:** | | Human | | **Number of entries searched:** | | 20266 | | **Specified cleavage agent(s):** | | Trypsin | | **Cleavage agent rule:** | | Trypsin (Preferentially cleaves at Arg and Lys in position C-terminal with higher rates for Arg) | | **Allowed number of missed cleavages:** | | 1 | | **Permissible amino acids modifications:** | | Fixed modification: Carbamidomethyl (C)  Variable modifications: Oxidation (M) | | **Precursor-ion mass tolerance for tandem MS:** | | 80-100  parts per million | | **Fragment-ion mass tolerance for tandem MS:** | | 0.3  dalton | | **Mass tolerance for PMF and other MS queries:** | | 50  parts per million | | **Thresholds; minimum scores for peptides, proteins:** | | p value lower than 0.05 | | **Selected scoring scheme in the software** | | Mowse scoring algorithm (MASCOT) | | **2.2.1 Additional parameters** | | **Maximum Peptide Rank** = 2 | |  | | **Max. number of MS peaks** = 65 | |  | | **Max. number of MSMS peaks** = 65 | |  | | **2.2.2 Database queried** | | **Name:** | | Database SwissProt taxonomy restringed to human | | **Version:** | | 57.15 | | **Number of sequences:** | | 20266 | | | --- | --- | --- | --- | --- | --- | --- | --- | --- | --- | --- | --- | --- | --- | --- | --- | --- | --- | --- | --- | --- | --- | --- | --- | --- | --- | --- | --- | --- | --- | --- | --- | --- | --- | --- | --- | --- | --- | --- | --- | --- | --- | --- | --- | --- | --- | --- | --- | --- | --- | --- | --- | --- | --- | --- | --- | --- | --- | --- | --- | --- | --- | --- | --- | --- | --- | --- | --- | --- | --- | --- | --- | --- | --- | --- | --- | --- | --- | --- | --- | --- | --- | --- | --- | --- | | | --- | --- | --- | --- | --- | --- | --- | --- | --- | --- | --- | --- | --- | --- | --- | --- | --- | --- | --- | --- | --- | --- | --- | --- | --- | --- | --- | --- | --- | --- | --- | --- | --- | --- | --- | --- | --- | --- | --- | --- | --- | --- | --- | --- | --- | --- | --- | --- | --- | --- | --- | --- | --- | --- | --- | --- | --- | --- | --- | --- | --- | --- | --- | --- | --- | --- | --- | --- | --- | --- | --- | --- | --- | --- | --- | --- | --- | --- | --- | --- | --- | --- | --- | --- | --- | --- | |  |  |
